# Bayesian versus diagnostic information in physician-patient communication: Effects of direction of statistical information and presentation of visualization

**DOI:** 10.1371/journal.pone.0283947

**Published:** 2023-06-07

**Authors:** Sarah Frederike Brose, Karin Binder, Martin R. Fischer, Martin Reincke, Leah T. Braun, Ralf Schmidmaier

**Affiliations:** 1 Department of Internal Medicine IV, University Hospital, LMU Munich, Munich, Germany; 2 Institute of Medical Education, University Hospital, LMU Munich, Munich, Germany; 3 Institute of Mathematics, LMU Munich, Munich, Germany; Universidad Rey Juan Carlos, SPAIN

## Abstract

**Background:**

Communicating well with patients is a competence central to everyday clinical practice, and communicating statistical information, especially in Bayesian reasoning tasks, can be challenging. In Bayesian reasoning tasks, information can be communicated in two different ways (which we call *directions of information*): The direction of *Bayesian information* (e.g., proportion of people tested positive among those with the disease) and the direction of *diagnostic information* (e.g., the proportion of people having the disease among those tested positive). The purpose of this study was to analyze the impact of both the direction of the information presented and whether a visualization (frequency net) is presented with it on patient’s ability to quantify a positive predictive value.

**Material and methods:**

109 participants completed four different medical cases (2⨯2⨯4 design) that were presented in a video; a physician communicated frequencies using different directions of information (Bayesian information vs. diagnostic information). In half of the cases for each direction, participants were given a frequency net. After watching the video, participants stated a positive predictive value. Accuracy and speed of response were analyzed.

**Results:**

Communicating with Bayesian information led to participant performance of only 10% (without frequency net) and 37% (with frequency net) accuracy. The tasks communicated with diagnostic information but without a frequency net were correctly solved by 72% of participants, but accuracy rate decreased to 61% when participants were given a frequency net. Participants with correct responses in the Bayesian information version without visualization took longest to complete the tasks (median of 106 seconds; median of 13.5, 14.0, and 14.5 seconds in other versions).

**Discussion:**

Communicating with diagnostic information rather than Bayesian information helps patients to understand specific information better and more quickly. Patients’ understanding of the relevance of test results is strongly dependent on the way the information is presented.

## Introduction

### Physician-patient communication

An appropriate manner of communicating that allows for shared decision-making with the patient is important no matter what specific field a physician works in [1]. Physicians can use different communication styles: physician-centered communication, patient-centered communication, and patient-centered communication with need-orientation. These have different effects on the patients’ evaluation of physicians and the information processing. In patient-centered communication, in contrast to physician-centered communication, the physician is perceived as more empathetic and socially as well as professionally competent [2]. It is effective for conveying factual information [2] and is often preferred [3]. Patient-centered communication with need-orientation is associated with lower knowledge acquisition [2] but is particularly suitable for patients who are aware of their personal needs [4] and when a good relationship needs to be established [2]. Patients with high trait anxiety, on the other hand, are often favorably disposed toward physician-centered communication [5]. Similarly, scientific versus emotional wording differentially influence physician-patient communication [6]. Thus, the appropriate use of particular communication styles depends on the specific patient groups and the consultation goals. In addition, it has been shown that patients tend to follow the physician’s advice and decide against their own initial treatment preferences [7–9]. In situations where patient preferences are of great importance, recommendations should be used carefully [10]. If the original preference is already in line with the physician’s recommendation, the patient’s decision-making certainty and satisfaction increases [9]. Good communication gives physicians the opportunity to pass on their knowledge about a specific disease as well as diagnostic and therapeutic considerations to their patients; it is essential for this information to be understood correctly by the patient. In clinical reality, the expectation that communication between physician and patient will be good is not always met [11]. Inadequacies are particularly apparent both in the making of a diagnosis and in its subsequent communication to the patient, as well as in communicating chances and risks of diagnostic methods or medical treatments [12, 13]. However, there are effective facilitating materials in the form of fact boxes or icon boxes for some encounters (screening tests, vaccinations) [14–16]. Situations which can be especially problematic are what is known as *Bayesian reasoning situations* because physicians themselves often struggle in estimating the correct positive predictive value [17, 18], and when the data is difficult for the physician, it is even more difficult for that physician to communicate the data in a Bayesian situation correctly and in a way that the patients will understand. In that context, there have been several studies (especially regarding HIV testing), that investigated what kind of information was being communicated by medical counselors to patients, and if that communicated information was correct or incorrect [13, 19, 20]. Furthermore, one prior study demonstrated that medical students can better distinguish between adequate and inadequate communication after having had Bayesian reasoning training (without directly being trained in communication skills; [21, 22]). In the present study, though we also focus on Bayesian reasoning problems, we specifically look at the influence of different ways of communicating statistical information on the ability of patients to deduce the positive predictive value.

### Bayesian reasoning in medicine

Many people assume that if a medical test has come back positive, it is highly probable that the patient has the disease. However, this probability depends not only on the sensitivity and the false-positive rate of the diagnostic test chosen but also on the prevalence of the disease. The *positive predictive value* is the probability that a patient with a positive test result actually has the disease. In clinical practice, it is understandably common that when a test result is positive (T+), the patient wants to know what this positive test result means. Therefore, the positive predictive value is the focus of the present study. If the sensitivity (*P*(*T*+│*D*)) and false-positive rate (i.e., 1 –specificity = *P*(*T*+│¬*D*)) of the test and the prevalence of the disease (*P*(*D*)) (or 1 –prevalence = *P*(¬*D*)) are known, the positive predictive value (*P*(*D*│*T*+)) can be calculated using Bayes’ formula [23]:

$$P\left(D|T+\right)=\frac{P\left(T+|D\right)P\left(D\right)}{P\left(T+|D\right)P\left(D\right)+P\left(T+|\neg D\right)P\left(\neg D\right)}$$

Bayes’ theorem is a powerful tool for medical decision-making [24] but often leads to cognitive overload [25]. Medical students, medical staff, and physicians often fail to calculate, for example, the positive predictive value of a mammography [26]. This statistical illiteracy can, of course, also be seen in the general population [27]. As a result, many patients overestimate their risk of a disease [28]. The consequence of an incorrect understanding of statistics can be overdiagnosis and overtreatment [29], with patients being the ones who suffer [30] as well as the society through the resulting costs in the health care system. Therefore, physicians should be able to explain to their patients what a positive test result actually means. This study aims to determine if certain ways of communicating statistical information can lead to better patient understanding of probabilities.

### Strategies to improve the understanding of statistical information

#### Information format

Statistical information can be specified using different formats, such as probabilities (e.g., 80%) or what is known as “natural frequencies” (e.g., 80 out of 100 people; see, e.g., [31, 32]). In Bayesian tasks, natural frequencies are recognized as enhancing understanding, and their use is therefore recommended [12, 33, 34]. With the help of natural frequencies, subjects can achieve higher solution rates [31, 35, 36] and arrive at task solutions more quickly [18, 37]. This positive effect has been seen in other studies in both (school and university) students [33, 34, 38] and physicians [17]. Accordingly, variation in information formats was not used in this study; only natural frequencies were used.

#### Direction of the information presented

Regardless of format, statistical information can be communicated in two different ways, which we call *directions of information* in the following. These directions of information are: *Bayesian information* and *diagnostic information*. Typically, in medical school and medical education, information is presented as Bayesian information (see Table 1, left). Bayesian information presents the proportion of individuals with a disease (prevalence), the proportion of individuals with conspicuous/positive testing when a disease is present (sensitivity), and the proportion of individuals with conspicuous/positive testing when a disease is not present (false-positive rate). Physicians and patients are frequently confronted with these data in clinical practice. An example is displayed in Table 1:

**Table 1:** Set of Bayesian information vs. diagnostic information in a typical Bayesian reasoning task.

Bayesian information	Diagnostic information
*Out of 1000 patients*, *50 patients have thyroid cancer*.	*Out of 1000 patients*, *130 patients have a conspicuous sonographic finding*.
*Of these 50 patients diagnosed with thyroid cancer*, *20 patients have a conspicuous sonographic finding*.	*Of these 130 patients with a conspicuous sonographic finding*, *20 patients actually have thyroid cancer*.
*On the other hand*, *of 950 patients who do not have thyroid cancer*, *110 patients still have a conspicuous sonographic finding*.	*On the other hand*, *of 870 patients with an inconspicuous sonographic finding*, *30 patients still have thyroid cancer*.

The Bayesian information here corresponds to the (Bayesian) tree diagram displayed in Table 1 (above). To answer the question of how many patients with a conspicuous sonographic finding actually have thyroid cancer, one must first calculate the number of patients with a conspicuous sonographic finding: 20 + 110 = 130 patients. The number of patients who have both a conspicuous test result and cancer directly appears in the task: 20 patients. Thus the question can be answered correctly as follows: 20 out of 130 patients with a conspicuous sonographic finding have thyroid cancer.

**Fig 1:**
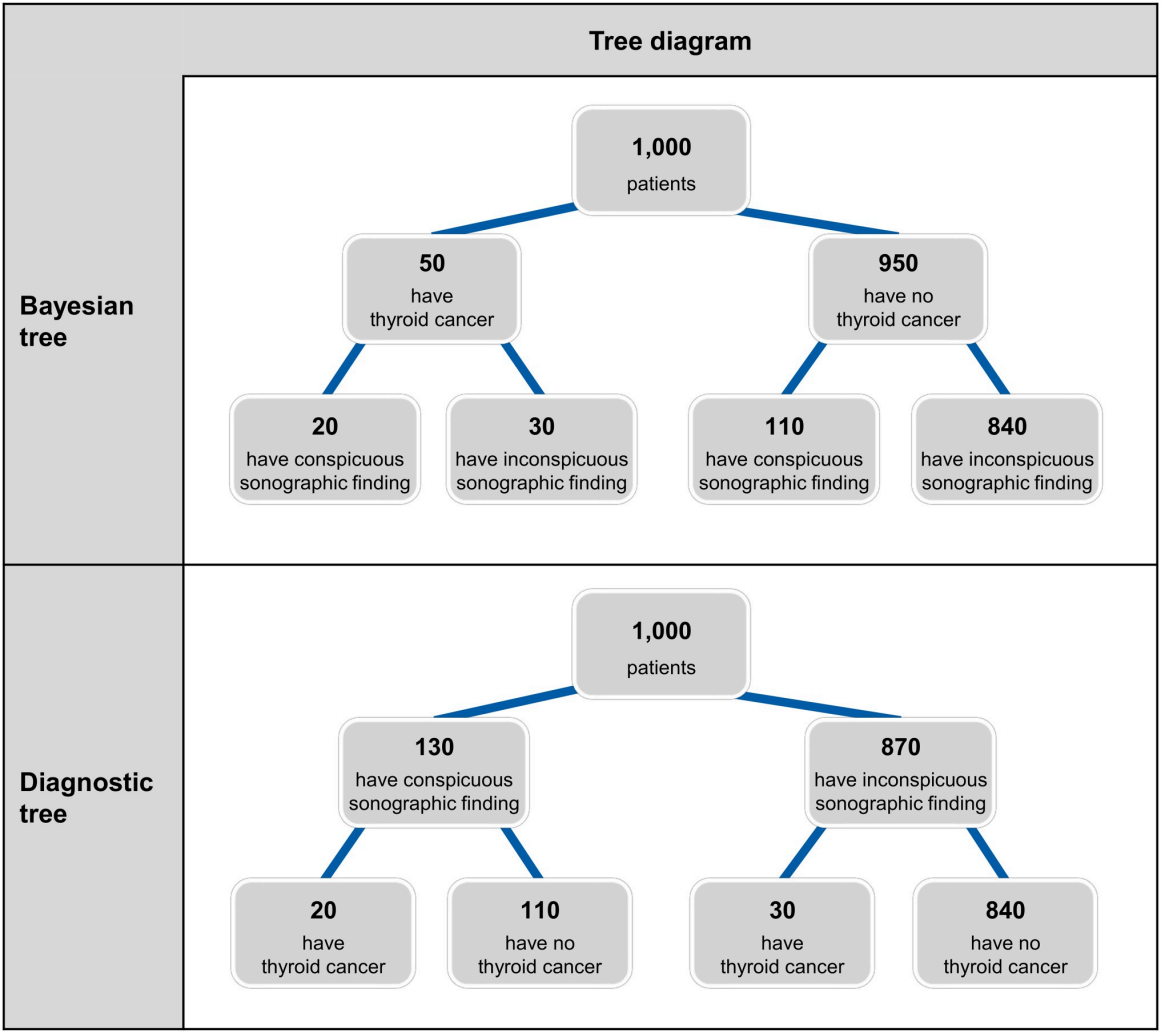
Bayesian tree vs. diagnostic tree. The statistical information displayed corresponds to the Bayesian information vs. diagnostic information presented in Table 1.

On the other hand, it is possible to provide statistical information in an inverted direction, which we call diagnostic information (see Table 1, right side). This set of information corresponds to what is known as a “diagnostic tree” (see Fig 1; [39, 40]). Diagnostic information involves the proportion of individuals with a conspicuous test, the proportion of individuals, who actually have the disease when there is a conspicuous/positive test (positive predictive value), and the proportion of individuals, who have the disease, despite an inconspicuous test (false-negative rate). Here, the question of positive predictive value can be answered directly without calculating. The answer remains the same as in the first example: 20 patients out of 130 patients with a conspicuous sonographic finding have thyroid cancer.

In an impressive study examining communication in HIV counseling centers, Prinz et al. [19] showed that about half of physicians are able to correctly name sensitivity and specificity, but hardly anyone can tell the patient the positive predictive value. In the present study, the focus is on *patients*, and we address the following question: What is the difference in patient understanding (regarding the positive predictive value) when physicians communicate with Bayesian information as opposed to when they communicate with diagnostic information?

#### Information visualization

In addition to the effect of the information format (natural frequencies vs. probabilities), it has been shown that diagnostic errors in Bayesian tasks can be reduced with the help of different *visualizations*, such as 2⨯2 tables, unit squares [41], icon arrays [42, 43], tree diagrams (Bayesian trees or diagnostic trees; see, e.g., Fig 1), double trees [38], or frequency nets [38]. Their use leads to an increase in solution rates [36, 44, 45] and also to a reduction of time needed on the task [18, 37].

**Fig 2:**
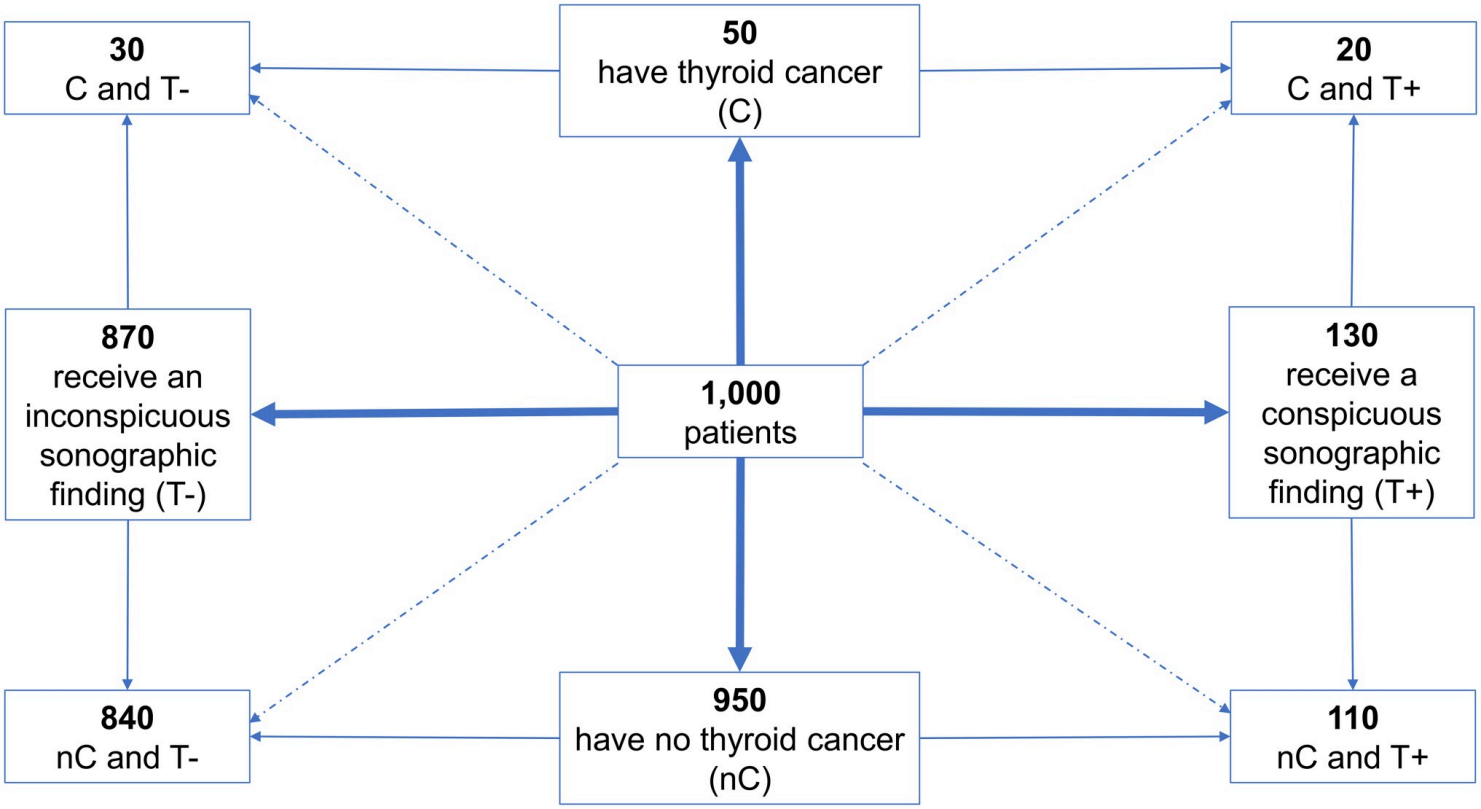
Frequency net for the thyroid cancer case.

The frequency net represents a relatively new visualization option that can help one to better understand probabilities and frequencies. This type of visualization was used in the present study. The frequency net has (e.g., compared to a typical Bayesian tree) the advantage that it makes visual both information directions—Bayesian information *and* diagnostic information—equally. It has already been shown that the frequency net increases the solution rate in Bayesian tasks compared to an explanation of probabilities that uses only text [38]. According to current research, the frequency net works well as a means of simplifying Bayesian reasoning since the visualizations allow the frequencies to be read off of the chart rather than having to be calculated. Fig 2 shows a frequency net for the thyroid cancer problem described above.

### Purpose of the study

The purpose of this study was to investigate the influence of the direction of communicated information presented (Bayesian information vs. diagnostic information) and visualization (without visualization vs. frequency net) on *solution accuracy (for the positive predictive value)* and the *solution speed* of potential patients in a physician-patient communication via video. We hypothesized that it is more helpful for participants if the physician communicates diagnostic information rather than Bayesian information because the diagnostic information set already includes the positive predictive value, whereas in the Bayesian information set, the correct solution is only displayed in the version that uses the frequency net.

Consequently we hypothesized that with Bayesian information, the version that uses the additional presentation of a frequency net (which actually contains diagnostic information) helps participants in answering the question that asks for the positive predictive value. The additional representation of a frequency net (which contains *both* sets of information) supplements the Bayesian information with the diagnostic information. On the other hand, when the primary information communicated is diagnostic, the results are exactly the opposite: When the diagnostic information set is used, the additional presentation of the frequency net supplements the already presented diagnostic information with Bayesian information. Therefore, the addition of the frequency net might be confusing, because there are many other probabilities displayed in it than simply the positive predictive value, and that extra information might in fact be *confused* with the positive predictive value, which might thus increase the cognitive load [46]. On the other hand, Mousavi et al. suggest that mixing auditory (i.e., the communicated diagnostic information from the physician) and visual (i.e., the frequency net) presentation modes reduces the cognitive load [47]. Because of these two contradictory influencing factors, we were interested, trying to determine which of the two factors predominates.

## Materials and methods

### Participants

In all, 110 participants took part in the study between August 2021 and June 2022 as patient proxies. However, since one participant processed only the introductory section and not the cases, only the results of the remaining 109 participants were analyzed. An age of at least 18 years was a prerequisite. There were no further inclusion or exclusion criteria, as anyone could potentially be a patient. Of these, 71% were female and 29% were male. The mean age was 29.2 years (*SD* = 11.2 years). Half of the participants had a high school diploma as their highest educational qualification, and about 39% had a university degree; 19% of the participants studied medicine or were employed in the medical field, and 11% of the participants studied or worked in the field of mathematics. Recruitment was done with the help of study advertisements. The study announcements were distributed via social media as well as hung up as flyers in the city of Munich in front of universities, libraries and hospitals. A certain bias can be assumed, since medical staff, students and younger people were particularly targeted. Participation places were distributed on a first come first serve basis. The task order was varied systematically, with each task order variant being used a similar number of times, to be able to control for any sequence effect (i.e., learning effects but also fatigue effects). Prior knowledge was not required, and only the title of the study—"Communication of statistical information to patients"—was communicated to interested participants in advance. Heterogeneity of the participants and random selection was desired, since a wide range of patients is also found in everyday clinical life. Even if patients in clinics are often older, there are also younger patients who are themselves ill or are accompanying older relatives who are ill. Therefore, the age for participation in the study was not further limited, except for an age of at least 18 years. Similarly, a professional background in the fields of medicine or mathematics was not a restriction, since everyday patients can range from laypeople to experts in the medical world. Choosing participants who actually had the respective diseases was refrained from since they might be strongly distracted by the content and influenced emotionally in their responses. Participation was voluntary and anonymous. The study was approved by the ethics commission of the LMU Munich (project number: 21–0024). All participants gave written informed consent and received a re-numeration of 10 euros for participation. Participation was remote and could be done from home using a computer with internet access. We carried out a pilot study with five participants to assess and improve the design of the study. The cases were solved in a reasonable amount of time (about 30–45 minutes). The layout was optimized.

### Study design

**Fig 3:**
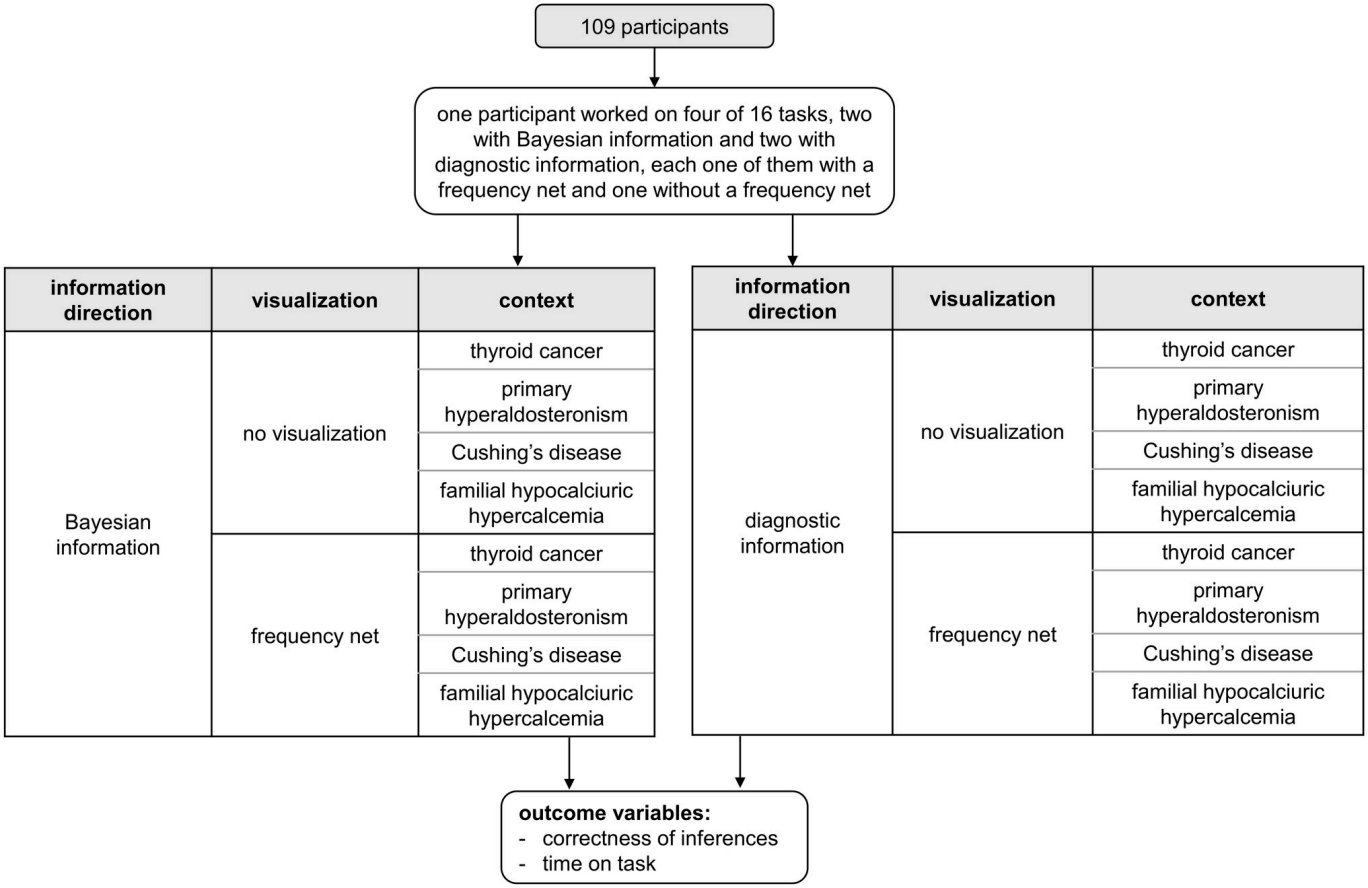
Study design.

The study was based on a 2⨯2⨯4 design as shown in Fig 3. The task versions varied in two different *directions of information presented* (Bayesian information vs. diagnostic information), *two different visualizations* (no visualization vs. frequency net), and *four different contexts* (thyroid cancer, primary hyperaldosteronism, Cushing’s disease, and familial hypocalciuric hypercalcemia; however, context was not a factor of interest in the study).

All contexts were from the endocrinology field and were derived from realistic clinical scenarios. They included details of various diseases and the test required for diagnosis, with the corresponding frequencies of occurrence taken from the current literature. All four different contexts are shown in S1–S4 Tables in the supporting information. Eight different task order variants were created, systematically varying the context, direction of information presented, and visualization. These different variants were randomly assigned to the participants, with each variant being used a similar number of times. The videos used in this study are available in German: https://www.doi.org/10.17605/OSF.IO/FZNWG

### Study process

**Fig 4:**
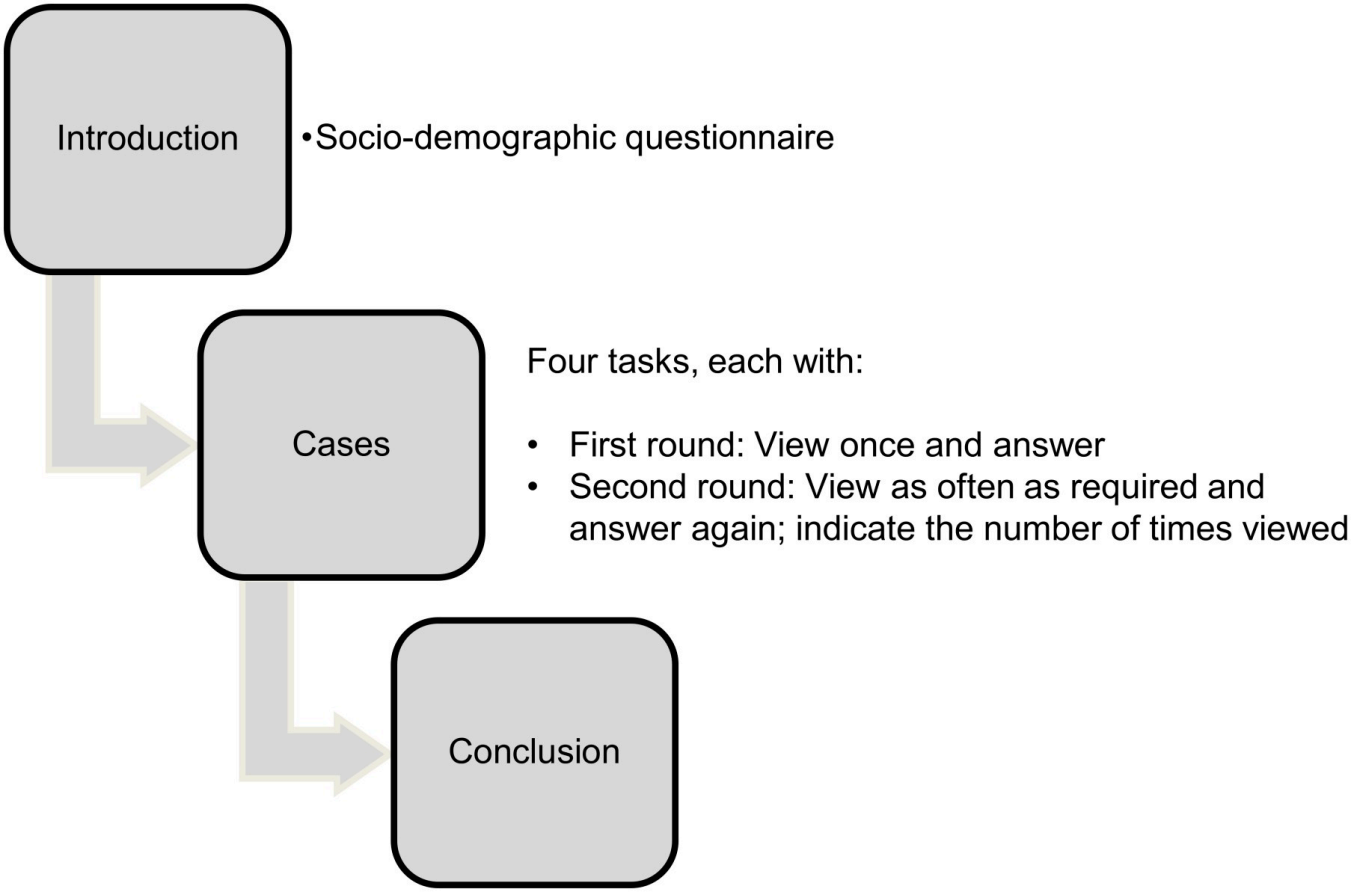
Study process.

The online learning platform CASUS (https://lmu.casus.net) was used to conduct the study. Each participant processed tasks for each context, two presented with Bayesian information and two with diagnostic information, and both given with and without visualization. The order of versions and contexts was systematically varied. The processing sequence was strictly predetermined so that any learning or fatigue effects could be controlled. The use of a calculator was allowed but was not necessary to solve the tasks. The study process can be seen in Fig 4. First, each participant read an introductory text and completed a general socio-demographic questionnaire (gender, age, highest level of education, current profession, self-assessment of basic medical knowledge, and self-assessment of basic mathematical knowledge as potential control variables that might influence participants’ performance). At the beginning of each video task, there was textual information that informed the participant that a video was to be shown in a moment, and also what question was going to be asked about the contents of that video at its completion. This approach is ecologically valid in the sense that in a real physician-patient interaction, the patient might ask a question *before* the physician provides the relevant information. Next, the video was shown of a physician-patient conversation in which a physician explains one of the contexts with the corresponding natural frequencies to a patient. After one viewing, measurement of time was started and participants were asked to answer the positive predictive value question, "How many people with a conspicuous/positive test result have disease x?" This involved inserting the two numbers asked into a cloze ("gap" patients out of "gap" patients). The time for completing the tasks was unlimited but recorded. In a second round, participants were allowed to watch the video as often as needed for them to feel confident in their answer, then were asked to state the answer, and finally indicate how many times they had watched the video prior to that answer. Seven participants did not indicate the frequency of watching the video, so that only the data of the remaining participants were used to evaluate the frequency.

### Statistical analysis

The results were binary coded. Responses were considered correct if both numbers asked for were given correctly (for example, in solving the thyroid cancer context "20 patients out of 130 patients," both numbers—"20" and "130"—had to be given). All analyses were conducted using R statistical software [48]. Statistical analyses were conducted using generalized linear mixed regression models and the package “lme4” [49].

Generalized linear mixed models (with a logit link function) were used to predict 1) participants’ performance in the tasks, and linear mixed models were used to predict 2) the time taken to solve the task. In this model, we specified the direction Bayes information and the version *without visualization* as the reference category and included the potential explanatory factors diagnostic information and frequency net via dummy coding.

## Results

### Accuracy of responses

#### Participants’ performance when analyzing the first task

**Fig 5:**
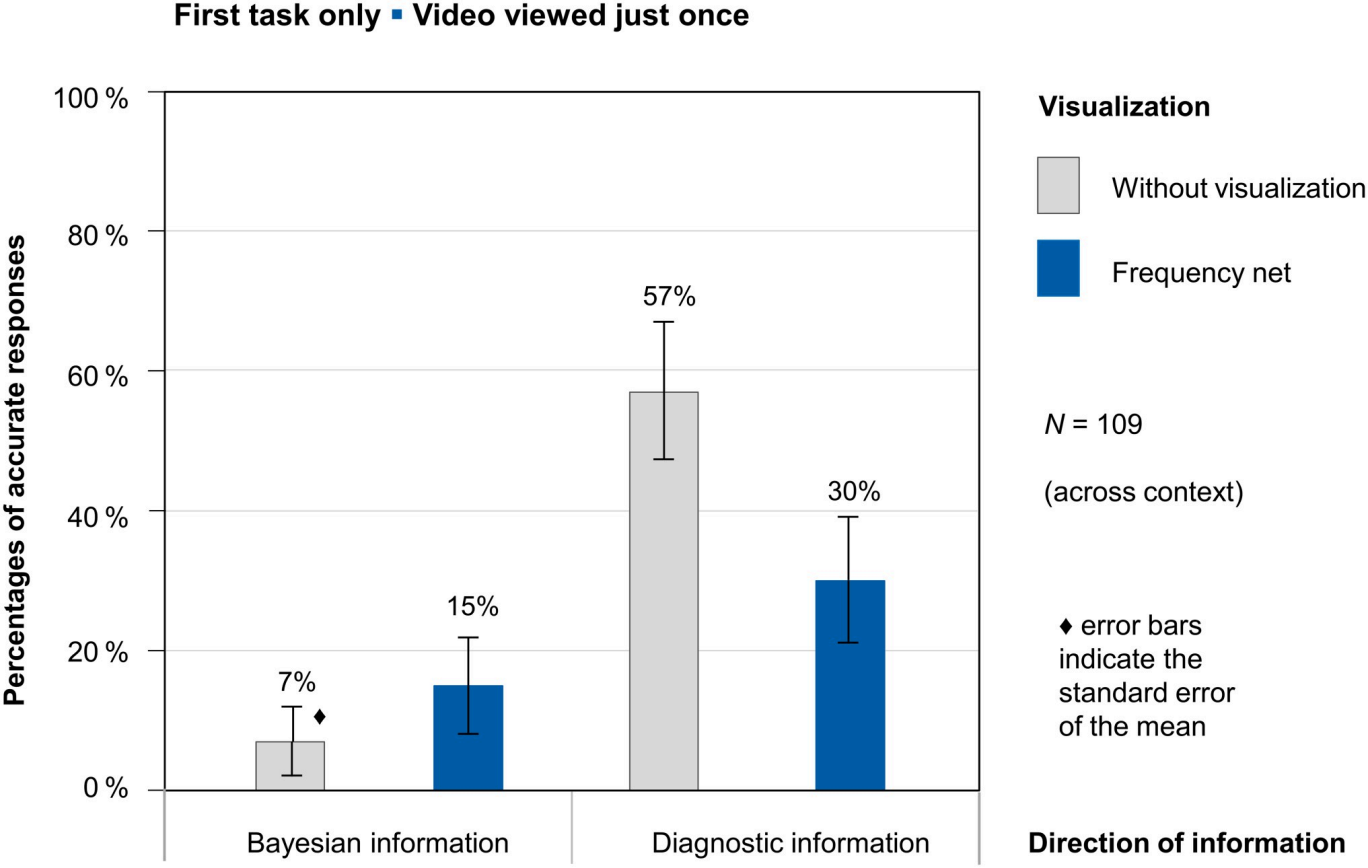
Performance of *N* = 109 participants after video was viewed just once; only first task analyzed. Errors bars indicate the standard error.

Fig 5 shows participants’ performance when only the *first task* administered to each participant is considered, and the response that the participant gave after the *first* viewing of the video. Thus any learning effects may not yet have occurred here.

As can be seen in Fig 5, there were large differences in results for the different versions. Only 7% of the participants were able to solve the task with Bayesian information and without visualization. Remember: Presenting a frequency net in the version with Bayesian information means additionally depicting diagnostic information, and 15% of participants were able to solve the task with Bayesian information when a frequency net (and therefore also the diagnostic information) was presented. However, case presentations communicated with diagnostic information showed descriptively higher solution rates after participants had watched the video only once. Participants’ performance was best in the diagnostic information version without a visualization (57%). The additional presentation of a frequency net (and therefore the additional presentation of Bayesian information) resulted descriptively in a reduction of the solution rate to 30%.

#### Participants’ performance when all tasks were analyzed and when multiple viewings were allowed

**Fig 6:**
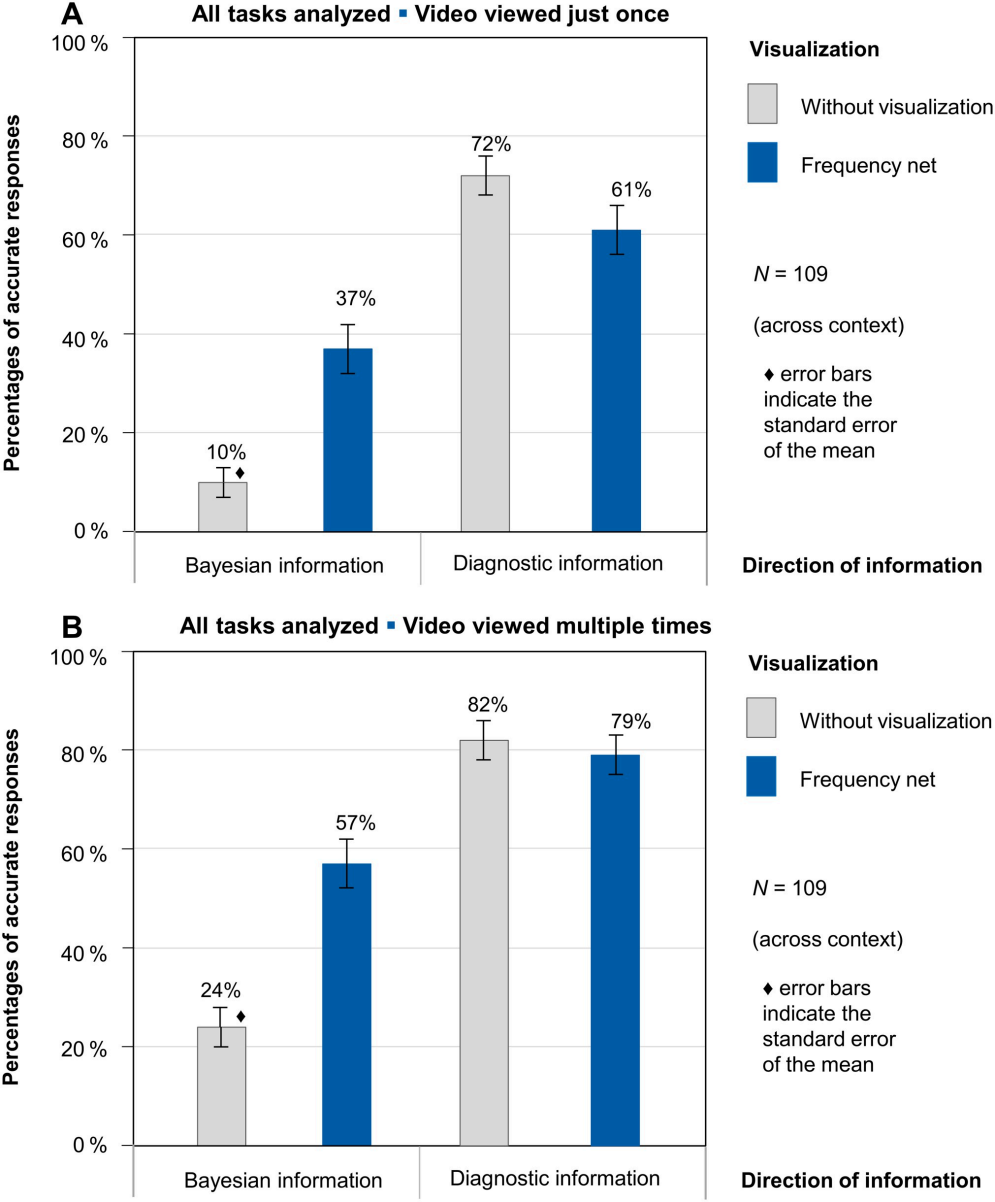
A. Performance of *N* = 109 participants after video was viewed just once; all tasks analyzed. Errors bars indicate the standard error. B. Performance of *N* = 109 participants after video was allowed to watch again; all tasks analyzed. Errors bars indicate the standard error.

When participant performance over all four tasks solved by each participant (Fig 6A) is considered, as well as participant performance when multiple viewings of the videos were allowed (Fig 6B), one important finding emerges. In the first instance, performance clearly becomes better as the participant progresses through the four tasks (comparing the percentages of correct responses in Figs 5 and 6A), and performance is also better after more than one viewing of the video (comparing the percentages of correct responses in Fig 6A and 6B), both of which indicate a learning effect for the participants (see also the generalized linear mixed model in the inferential statistical analyses).

If all tasks are included in the analysis, descriptively higher solution rates can be observed, since the task that was worked on first was on average the one with the worst performance levels. Averaging participants’ performance across all tasks increased from 7% to 10% in the Bayesian information version without a visualization. With the help of a frequency net (which adds diagnostic information), 37% of the participants (as compared to 15%) were able to solve a Bayesian information task; 72% of the participants (as compared to 57%) were able to solve a diagnostic information task without visualization; and 61% (as compared to 30%) of the participants were able to solve a diagnostic information task with the help of the frequency net. Thus there is descriptively a sequence effect in participants’ performance. Tasks that were performed later in the sequence of the four tasks were solved with more accuracy. It must be noted again that tasks with Bayesian information and diagnostic information were processed alternately (that is to say, in half of the tasks the Bayesian information was shown first, and in the other half of the tasks the diagnostic information was shown first).

#### Generalized linear mixed model for predicting participants’ performance after the video was viewed only once

In order to statistically compare the effects of *direction of information* and *frequency net*, we estimated a generalized linear mixed model with a logit link function to predict *participants’ performance after the video was only viewed once*, *but across all tasks*. In this model, we specified the Bayesian information direction without the frequency net as the reference category and included the possible explanatory factors “diagnostic information” and “frequency net” via dummy coding. In addition, since the effectiveness of the frequency net was expected to differ depending on whether Bayesian or diagnostic information was shown, an interaction term *direction of information ⨯ frequency net* was modeled. Furthermore, age, gender, and the highest level of education were collected from all participants. These variables, and also the context of the task (which disease or syndrome) and the sequence of the task (i.e., was the task provided as the first, second, third, or fourth task) could be implemented as potential predictors in the generalized linear mixed model. In the following we present the results of the following model

$$\text{Y}={\text{b}}_{0}+{\text{b}}_{1}\cdot \text{direction}\;\text{of}\;\text{information}+{\text{b}}_{2}\cdot \text{frequency}\;\text{net}+{\text{b}}_{3}\cdot \text{direction}\;\text{of}\;\text{information}\times \text{frequency}\;\text{net}+{\text{b}}_{4}\cdot \text{sequence}\;\text{of}\;\text{the}\;\text{task}+{\text{b}}_{5}\cdot \text{age}+{\text{b}}_{6}\cdot \text{gender}$$

with a logistic link function $\frac{e^{Y}}{1+e^{Y}}$.

As can be seen from Table 2, the regression coefficient for reversing the direction of statistical information from Bayesian to diagnostic information was significant, and presenting a frequency net also led to a significant regression coefficient. As already expected and descriptively supported, the effectiveness of the frequency net depends on whether Bayesian information or diagnostic information is presented: The interaction effect between direction and visualization is significant. Thus, the effect of the frequency net decreases significantly when diagnostic information is shown instead of Bayesian information. As the descriptive results had already indicated, there is a significant effect associated with the sequence of the task (i.e., was the task provided as the first, second, third, or fourth task). Another statistical significant predictor is the age of the participant (the younger the participant in our study, the better the overall performance). In contrast, other variables (e.g., context, gender) were not significant factors of influence.

**Table 2:** Parameter estimates from generalized linear mixed model for predicting participants’ performance after having seen the video once.

Covariates	Estimate	*SE*	*z*	*p*
Intercept	-0.53	0.25	-2.13	**0.03**
Direction of information	1.77	0.22	7.97	**<0.001**
Frequency net	0.46	0.16	2.94	**0.003**
Direction of information ⨯ frequency net	-0.87	0.17	-5.08	**<0.001**
Sequence of the task	0.69	0.16	4.24	**<0.001**
Age	-0.62	0.25	-2.42	**0.02**
Gender	0.32	0.25	1.29	0.20

*Remark*: Other possible covariates (such as context of the task (via dummy coding) or highest level of education) were not significant predictors (like gender was also no significant predictor in the model), when implementing in the model.

Multiple viewings of the videos improved performance—in all versions—even more, as can be seen in Fig 6B. In summary, the following can be stated: The best performance is achieved when the statistical information is presented in the direction of diagnostic information rather than in the direction of Bayesian information. The frequency net (which augments the Bayesian information version with diagnostic information) is helpful when it is Bayesian information presented. However, the frequency net is more of a barrier than an aid if diagnostic information is what has been communicated (since the frequency net only complements Bayesian information). This negative effect of the frequency net when used with diagnostic information is descriptively reduced if the video has been viewed several times or if similar tasks have already been processed.

The number of viewings necessary for participants to be sure of the correct response also differs depending on the version being used: In tasks with diagnostic information, videos were viewed by the participants fewer times (on average 1.7 times in both versions, with or without the frequency net) than tasks presented with Bayesian information (average of 1.8 times with frequency net; average of 2.1 times without).

#### Generalized linear mixed model for predicting participants’ performance after having seen the *video multiple times*

We also specified another generalized linear mixed model, one in which the dependent variable was participants’ performance across all tasks, after the video was allowed to be viewed multiple times (see Table 3). Again, direction of information and the frequency net were implemented as independent variables via dummy coding. Additionally, the variables *age*, *gender*, *highest level of education*, *context*, *sequence of the task* and *how often the video was watched* could be implemented as potential predictors. In the following we present the results of the following model

$$\text{Y}={\text{b}}_{0}+{\text{b}}_{1}\cdot \text{direction}\;\text{of}\;\text{information}+{\text{b}}_{2}\cdot \text{frequency}\;\text{net}+{\text{b}}_{3}\cdot \text{direction}\;\text{of}\;\text{information}\times \text{frequency}\;\text{net}+{\text{b}}_{4}\cdot \text{sequence}\;\text{of}\;\text{the}\;\text{task}+{\text{b}}_{5}\cdot \text{age}+{\text{b}}_{6}\cdot \text{gender}+{\text{b}}_{7}\cdot \text{frequency}\;\text{of}\;\text{watching}\;\text{the}\;\text{video}$$

again with a logistic link function. As in the model before, the regression coefficient for reversing the direction of statistical information from Bayesian to diagnostic information was again significant, and presenting a frequency net also led to a significant regression coefficient. As before, the effectiveness of the frequency net depended on whether Bayesian information or diagnostic information was shown: The interaction effect between direction and visualization was significant. Thus the effect of the frequency net again decreased significantly when diagnostic rather than Bayesian information was presented. However, the variables sequence of task and age are no longer significant in this model. A learning effect could be observed resulting from two processes: Watching a video multiple times and learning from a task that was worked on before. Both lead to better performance, and with the use of the diagnostic information version, to the fact that the frequency net is no longer the barrier described earlier. Interestingly, the frequency of watching the video was not a significant factor in the model. Nevertheless, participants’ performances improved when they were allowed to correct their answers after watching the video several times (compare performance rates in Fig 6A and 6B). Presumably, participants in our study were not completely honest in answering the question of how often they watched the video, because many of them corrected the previously wrong answer after watching the video several times and were then able to give the correct answer.

**Table 3:** Parameter estimates from generalized linear mixed model for predicting participants’ performance after having seen the video multiple times.

Covariates	Estimate	*SE*	*z*	*p*
Intercept	0.80	0.27	2.95	**0.003**
Direction of information	1.69	0.23	7.40	**<0.001**
Frequency net	0.60	0.16	3.64	**<0.001**
Direction of information ⨯ frequency net	-0.74	0.17	-4.35	**<0.001**
Sequence of the task	0.15	0.16	0.92	0.36
Age	-0.52	0.27	-1.96	0.05
Gender	0.29	0.26	1.12	0.26
Frequency of watching the video	0.08	0.19	0.42	0.67

*Remark*: Other possible covariates (such as context of the task (via dummy coding) or highest level of education) were not significant predictors (like gender was also no significant predictor in the model), when implementing in the model.

### Time taken for responses

A similar picture emerges in terms of the median time taken by participants to answer the question after having watched the video once. Table 4 shows separate median times (and 25- and 75-quartiles) taken by participants for incorrect and correct responses. The first thing to notice is that *incorrectly* solved tasks take a similar amount of time to answer and seem not to depend so much on which version was used (the median ranges from 16.0 to 22.5 seconds for the different versions). In the *correctly* answered tasks, on the other hand, there is a clear difference in the completion times. The tasks with diagnostic information and the Bayesian information tasks with the frequency net are processed similarly quickly (median ranges from 13.5 to 14.5 seconds), but the version with Bayesian information and without visualization requires an increased completion time, with a median of 106 seconds, since the calculation of a correct solution is algorithmically more complex here. If Bayesian information is communicated with an additional frequency net (which also carries diagnostic information), the median completion time is much faster (median: 13.5 seconds). If we compare the times of our study with Kunzelmann et al. (2022) and Binder et al. (2021), we notice that they were significantly shorter. The reason for this is a different setting: In our study, it is the pure processing time for the question, which was started after reading the task and watching the video for the first time. In contrast, in the other studies, the reading time was also included in the time, which obviously takes up a considerable part of the solution process. Moreover, in the present study, the question was already known, so it was easier to focus on the information.

**Table 4:** Descriptive results across all contexts and across all solved tasks after the video was viewed once.

			Percentage of accurate responses	Time taken for an incorrect response				Time taken for a correct response				Diagnostic efficiency score: Median time taken for correct responses dived by percentage of correct responses
					[sec.]				[sec.]			time in [min:sec] / accuracy
		N	*%*	N	Q1	Median	Q3	N	Q1	Median	Q3	
**Bayesian information**	No visualization	109	10	98	12.0	22.5	42.0	11	13.0	106.0	133.0	17:30
	Frequency net	109	37	69	12.0	21.0	32.0	40	9.0	13.5	20.5	0:36
**Diagnostic information**	No visualization	109	72	31	12.0	16.0	26.0	78	10.0	14.5	19.0	0:20
	Frequency net	109	61	42	13.0	21.5	32.0	67	9.0	14.0	30.0	0:23

Percentage of accurate responses, time taken for an incorrect or a correct response with 1^st^ (Q1), median, and 3^rd^ (Q3) quartile; score for diagnostic efficiency. The diagnostic efficiency score was calculated by dividing the median time by the percentage of correct responses. N indicates the number of participants.

A linear mixed model to estimate the mean time needed to reach a correct response was modeled. The model shows that the direction of presented information (p < 0.001) and the presentation of a frequency net (p < 0.001) had a significant effect on the completion time for a correct response. Furthermore, there is a significant interaction effect of *direction of information ⨯ visualization* (p < 0.001), which means that the advantage seen with the use of the frequency net on completion time depends on whether it is Bayesian or diagnostic information being presented. Once again the sequence of tasks (i.e., was the task presented as the first, second, third, or fourth task; p < 0.001) can be seen to have a significant effect on the completion time. Also the context of Cushing’s disease caused participants to need significantly more time, women needing significantly more time than men, without a change in the reported effects of information direction or visualization. Other variables (such as age) had no significant effect on completion time.

Since both accuracy and time are crucial factors in communication, we provide a score that integrates both of these factors. Therefore, Table 4 shows the score *diagnostic efficiency*. The diagnostic efficiency score divides the median time on *correctly* solving a task by the proportion of correct responses made (alternatively, scores could also be calculated that divide the median time on *any* task by the proportion of correct responses; compare [18]). The best score for diagnostic efficiency was obtained with the use of the diagnostic information version without visualization (20 seconds), whereas the worst score was obtained in the Bayesian information version without visualization (17 minutes, 30 seconds).

## Discussion

We need more studies that make concrete suggestions on how to improve risk communication (see, e.g., [16]).

### Bayesian reasoning causes difficulties for patients

This study examined the influence of the direction of the information set presented (Bayesian information or diagnostic information) and visualization (none or with the frequency net) on patients’ ability to find the correct solution. Participants performed consistently better in versions using diagnostic information (as compared to Bayesian information). This result is also evident in the versions accompanied by a frequency net: Adding a frequency net to the Bayesian information direction in essence means showing additional diagnostic information to participants, which in turn was helpful. However, adding a frequency net to the diagnostic information direction means showing them additional Bayesian information, which in turn was *not* helpful to them.

In this study, statistical information was shown to cause problems for potential patients: Only 10% of participants were able to solve Bayesian tasks that were communicated purely auditorily. Yet the presence of Bayesian information is not uncommon in everyday medical practice, and being able to come to a correct and quick response is essential for physicians and patients. Particularly striking in this study was the time needed by participants to solve the Bayesian tasks correctly without any visualization provided. Since time is a limited resource in clinical settings, it is important to minimize the amount necessary for these kind of tasks. Presenting information in diagnostic form can address this problem. In the statistical analyses from this study, it was evident that, in addition to a shorter solution time, information presented in the diagnostic direction also allowed for more correct conclusions to be reached (72% in the version without visualization). The mechanism behind this greater level of success presumably is that the positive predictive value is already directly stated in the original data presented and does not have to be calculated as it does with Bayesian information. Consequently, diagnostic information communication might be more efficient in the clinical setting.

### The frequency net helps in understanding Bayesian information

As an alternative to auditory explanation, a frequency net was used for visualization in half of the tasks in this study. In Bayesian tasks, that use led to better understanding, as seen by a higher solution rate (37%), and these results are in line with those of other studies in which other visualization options were shown to support understanding of Bayesian situations [18, 33]. But the usefulness of a frequency net specifically has previously only been investigated in comparison with written explanations [38]; this study now extends that scope to comparison with auditory explanations. The reason for the ensuing better understanding with the use of a frequency net presumably is that in the tasks with visualizations, the frequency asked for can be read off of the diagram, whereas without a visualization, the Bayesian frequencies first have to be calculated before an answer can be given. The opposite was seen when diagnostic rather than Bayesian information was communicated: The number of correct solutions decreased by a small amount to 61% with the use of the frequency net. The reason for this drop could perhaps be cognitive overload produced by the additional presentation of Bayesian information to the diagnostic information, although we did not assess cognitive load. The additional frequencies presented in the frequency net seem to have led to confusion, while in the solely auditory versions, only the specific necessary information was conveyed [50].

### Improving physician-patient communication through diagnostic information presentation and targeted use of visualizations

According to the results of this study, diagnostic information communication is preferable to Bayesian information for reasons of efficiency. However, studies have shown that diagnostic information is often not communicated by physicians, but Bayesian information is (see, e.g., [19]). If, then, only Bayesian information is available, it could be converted by physicians or medical staff into diagnostic information prior to any meeting with the patient. If that is not possible, then visualization should be used as an adjunct in explaining the more complicated Bayesian information. In addition, communicating the frequencies at least several times would be helpful in the use of any version. In this study, it was evident that by communicating several different diagnostic situations in sequence, solution rates could be increased significantly (comparing the percentages of correct responses in Figs 5 and 6A and in Fig 6A and 6B). Putting effort into communicating statistical information is the only way to guarantee that patients understand the results of their tests correctly and that a reasonable, shared decision can be made between physician and patient on how to proceed. These guidelines could ultimately improve the important competence of physician-patient communication in the long term. To that end, we need even more studies that can make concrete suggestions on how to improve risk communication (as in [51]). In addition, medical students should be required to learn, as part of their studies, to convert statistical information in a patient-friendly way and create visualizations.

### Limitations and outlook

In this study the majority of the participants were young, female, and had completed a high level of education. In clinical practice, most patients are older, and all educational strata are represented. This discrepancy between real patients and study participants could have implications in terms of their understanding of Bayesian situations. Another limitation of the study is that the frequency net was used as a visualization. Therefore, the benefit of other visualizations can be assumed but not generalized. It would then be interesting to investigate further as to whether these results can be replicated when a frequency double tree, for example, is used. The next step would be to test the feasibility of implementing our recommendations for good communication into everyday clinical practice and applying them in that way.

## Conclusion

Understanding statistical information is highly dependent on how it is communicated. To enable patients to make good decisions, physicians should try to optimize several parameters in their communication. Diagnostic information should be communicated instead of Bayesian information, or Bayesian information should be communicated in combination with the presentation of a frequency net to increase the patient’s understanding of the overall situation. Furthermore, the changes in results in this study after multiple viewings of the videos suggest another influence: A very crucial factor in the patient’s understanding seems to be a deeper examination of the physician’s explanation (by multiple viewings of the videos). In our study, a more intensive examination of the situation helped participants to come to a correct solution. Furthermore, the learning effect shown in our study is gratifying: Patients are—with proper communication—quite capable of understanding the situation.

## Supporting information

S1 Table Thyroid cancer case formulations. (DOCX)

S2 Table Primary hyperaldosteronism case formulations. (DOCX)

S3 Table Cushing’s disease case formulations. (DOCX)

S4 Table Familial hypocalciuric hypercalcemia case formulations. (DOCX)

S1 Fig Frequency net for the thyroid cancer case. (TIF)

S2 Fig Frequency net for the primary hyperaldosteronism case. (TIF)

S3 Fig Frequency net for the Cushing’s disease case. (TIF)

S4 Fig Frequency net for the familial hypocalciuric hypercalcemia case. (TIF)
