# Supplementary material for: Bayesian versus diagnostic information in physician-patient communication: Effects of direction of statistical information and presentation of visualization
Source: PLoS One. 2023 Jun 7;18(6):e0283947. doi: 10.1371/journal.pone.0283947 (PMC10246784; doi:10.1371/journal.pone.0283947)
Supplement: S1 Table — (DOCX) [file pone.0283947.s001.docx]

|  | Version 1 | Version 2 | Version 3 | Version 4 |
| --- | --- | --- | --- | --- |
|  | Bayesian information | | Diagnostic information | |
| Introduction | You have just had a sonogram of your thyroid to confirm or rule out the presence of thyroid cancer. Now I would like to discuss your test results with you.  Unfortunately, I have to inform you that your test showed a conspicuous sonographic finding. I would now like to explain to you exactly what a conspicuous test result means.  (In this frequency net, 1000 patients were screened for the presence of a conspicuous sonographic finding as well as thyroid cancer. The two criteria of a conspicuous or inconspicuous sonographic finding and the presence of thyroid cancer or no thyroid cancer, are visualized here both individually and in combination). | | | |
| Information direction | - Out of 1000 patients, 50 patients have thyroid cancer. - Of these 50 patients diagnosed with thyroid cancer, 20 patients have a conspicuous sonographic finding. - On the other hand, of 950 patients who do not have thyroid cancer, 110 patients still have a conspicuous sonographic finding. | | - Out of 1000 patients, 130 patients have a conspicuous sonographic finding. - Of these 130 patients with a conspicuous sonographic finding, 20 patients actually have thyroid cancer. - On the other hand, of 870 patients with an inconspicuous sonographic finding, 30 patients still have thyroid cancer. | |
| Visualization | No visualization | Frequency net (S1 Fig) | No visualization | Frequency net (S1 Fig) |
| Question | How many patients with a conspicuous sonographic finding have thyroid cancer?  Answer: 20 out of 130 patients | | | |
