# Supplementary material for: Bayesian versus diagnostic information in physician-patient communication: Effects of direction of statistical information and presentation of visualization
Source: PLoS One. 2023 Jun 7;18(6):e0283947. doi: 10.1371/journal.pone.0283947 (PMC10246784; doi:10.1371/journal.pone.0283947)
Supplement: S2 Table — (DOCX) [file pone.0283947.s002.docx]

|  | Version 1 | Version 2 | Version 3 | Version 4 |
| --- | --- | --- | --- | --- |
|  | Bayesian information | | Diagnostic information | |
| Introduction | You have been diagnosed with hypertension. Yesterday, blood was taken from you to clarify the cause of your hypertension. The aldosterone-renin quotient was determined to confirm or rule out the suspicion of Conn’s syndrome. A conspicuous test result would be indicative of Conn’s syndrome. I have asked you here again today to discuss your test results with you.  Unfortunately, I have to inform you that your test showed a conspicuous aldosterone-renin quotient. I would now like to explain to you exactly what a conspicuous test result means.  (In this frequency net, 1000 patients with hypertension were examined for the presence of a conspicuous aldosterone-renin quotient as well as Conn’s syndrome. The two criteria of a conspicuous or inconspicuous aldosterone-renin quotient and Conn’s syndrome or no Conn’s syndrome are visualized here both individually and in combination). | | | |
| Information direction | - Out of 1000 patients, 80 patients have Conn’s syndrome. - Of these 80 patients diagnosed with Conn’s syndrome, 50 patients have a conspicuous aldosterone-renin quotient. - On the other hand, out of 920 patients who do not have Conn’s syndrome, 300 patients still have a conspicuous aldosterone-renin quotient. | | - Out of 1000 patients, 350 patients have a conspicuous aldosterone-renin quotient. - Of these 350 patients with a conspicuous quotient, 50 patients actually have Conn’s syndrome. - On the other hand, out of 650 patients with an inconspicuous result, 30 patients still have Conn’s syndrome. | |
| Visualization | No visualization | Frequency net (S2 Fig) | No visualization | Frequency net (S2 Fig) |
| Question | How many patients with a conspicuous aldosterone-renin quotient have Conn’s syndrome?  Answer: 50 out of 350 patients | | | |
