# Supplementary material for: Bayesian versus diagnostic information in physician-patient communication: Effects of direction of statistical information and presentation of visualization
Source: PLoS One. 2023 Jun 7;18(6):e0283947. doi: 10.1371/journal.pone.0283947 (PMC10246784; doi:10.1371/journal.pone.0283947)
Supplement: S3 Table — (DOCX) [file pone.0283947.s003.docx]

|  | Version 1 | Version 2 | Version 3 | Version 4 |
| --- | --- | --- | --- | --- |
|  | Bayesian information | | Diagnostic information | |
| Introduction | Recently, you had the 8 mg dexamethasone inhibition test. This is a test that can be used to distinguish whether your already confirmed hypercortisolism is Cushing's disease or ectopic Cushing's syndrome. For this, your blood was drawn on two consecutive mornings, with you having taken a tablet of 8-mg dexamethasone on the evening of the first day. A positive test result can indicate Cushing's disease. Today we would like to discuss your test results with you.  The test was positive. I would now like to explain to you exactly what a positive test result means.  (In this frequency net, 1000 patients with hypercortisolism were examined for a positive or negative result in the 8-mg dexamethasone inhibition test, as well as Cushing’s disease or ectopic Cushing's syndrome. The two criteria of test result and state of disease are presented in this visualization, both individually and in combination). | | | |
| Information direction | - Out of 1000 patients, 890 patients have Cushing's disease. - Of these 890 patients diagnosed with Cushing's disease, 720 patients have a positive result in the 8-mg dexamethasone inhibition test. - On the other hand, of 110 patients who have ectopic Cushing's syndrome, 40 patients still have a positive result from the 8-mg dexamethasone inhibition test. | | - Out of 1000 patients, 760 patients have a positive result in the 8 mg dexamethasone inhibition test. - Of these 760 patients with a positive result, 720 patients actually have Cushing's disease. - On the other hand, out of 240 patients with a negative result, 170 patients still have Cushing's disease. | |
| Visualization | No visualization | Frequency net (S3 Fig) | No visualization | Frequency net (S3 Fig) |
| Question | How many patients with a positive 8 mg dexamethasone inhibition test have Cushing's disease?  Answer: 720 out of 760 patients | | | |
