# Supplementary material for: Bayesian versus diagnostic information in physician-patient communication: Effects of direction of statistical information and presentation of visualization
Source: PLoS One. 2023 Jun 7;18(6):e0283947. doi: 10.1371/journal.pone.0283947 (PMC10246784; doi:10.1371/journal.pone.0283947)
Supplement: S4 Table — (DOCX) [file pone.0283947.s004.docx]

|  | Version 1 | Version 2 | Version 3 | Version 4 |
| --- | --- | --- | --- | --- |
|  | Bayesian information | | Diagnostic information | |
| Introduction | Yesterday you submitted a urine sample collected over 24 hours, which allowed the lab to determine the calcium/creatinine ratio in your urine to test whether you have familial hypocalciuric hypercalcemia or primary hyperparathyroidism. A positive test result would indicate familial hypocalciuric hypercalcemia. I have asked you here again today to discuss your test results with you.  The test was positive. I would now like to explain to you exactly what a positive test result means.  (In this frequency net, 1000 patients were examined for a positive or negative result in the calcium/creatinine ratio, as well as familial hypocalciuric hypercalcemia or primary hyperparathyroidism. The two criteria of the test result and the diseases are presented visually here, both individually and in combination). | | | |
| Information direction | - Out of 1000 patients, 20 patients have familial hypocalciuric hypercalcemia. - Of these 20 patients diagnosed with familial hypocalciuric hypercalcemia, all 20 patients have a positive result in the urinary calcium/creatinine ratio. - On the other hand, of 980 patients who have primary hyperparathyroidism, 250 patients still have a positive result in the calcium/creatinine ratio. | | - Out of 1000 patients, 270 patients have a positive result in the calcium/creatinine ratio. - Of these 270 patients with a positive result, 20 patients actually have familial hypocalciuric hypercalcemia. - On the other hand, out of 730 patients with a negative result, 3 patients still have familial hypocalciuric hypercalcemia. | |
| Visualization | No visualization | Frequency net (S4 Fig) | No visualization | Frequency net (S4 Fig) |
| Question | How many patients with a positive result from the calcium/creatinine ratio test have familial hypocalciuric hypercalcemia?  Answer: 20 out of 270 patients | | | |
