# Supplementary figures and images for: Bayesian versus diagnostic information in physician-patient communication: Effects of direction of statistical information and presentation of visualization
Source: PLoS One. 2023 Jun 7;18(6):e0283947. doi: 10.1371/journal.pone.0283947 (PMC10246784; doi:10.1371/journal.pone.0283947)

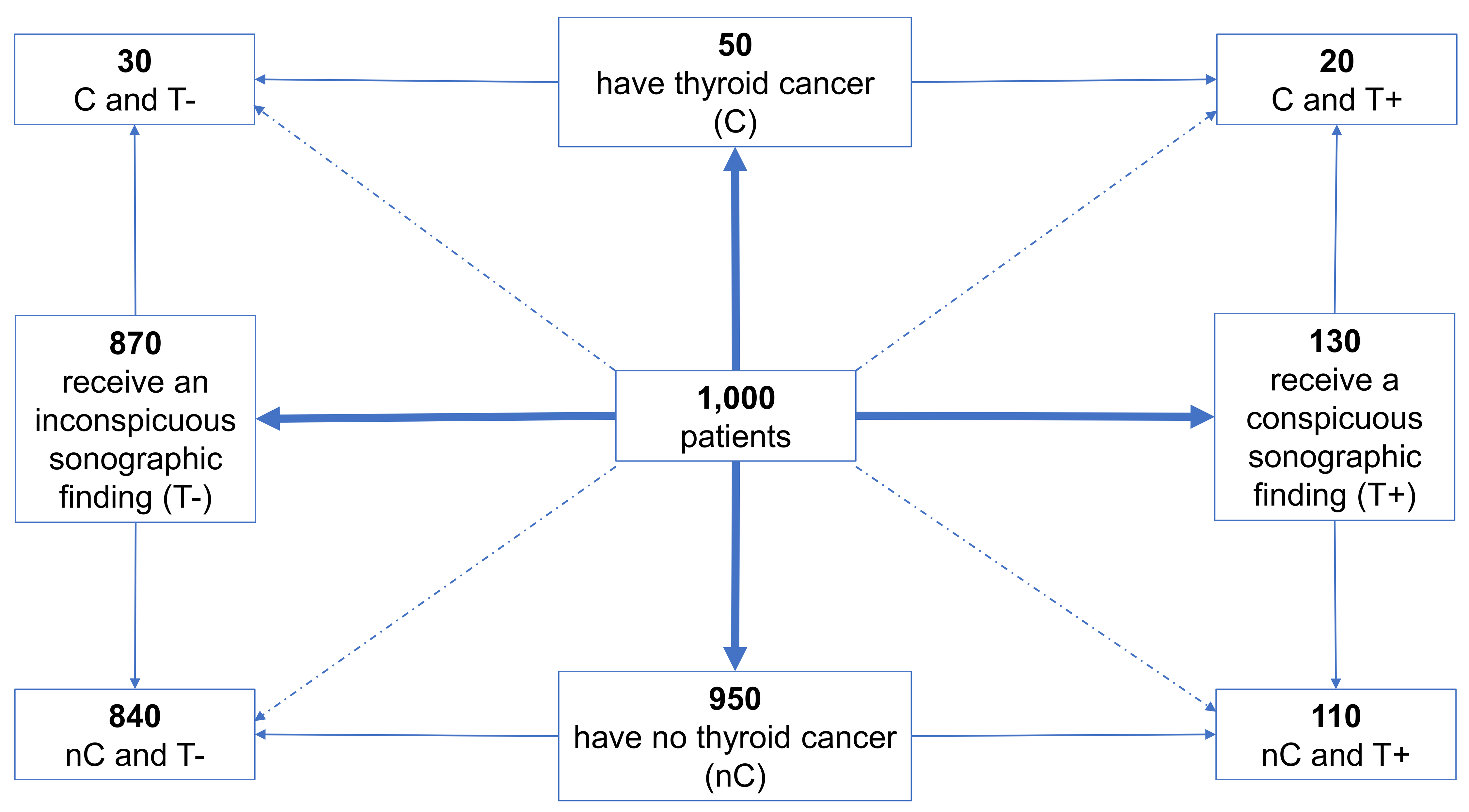

Supplement: S1 Fig — (TIF) [file pone.0283947.s005.tif]

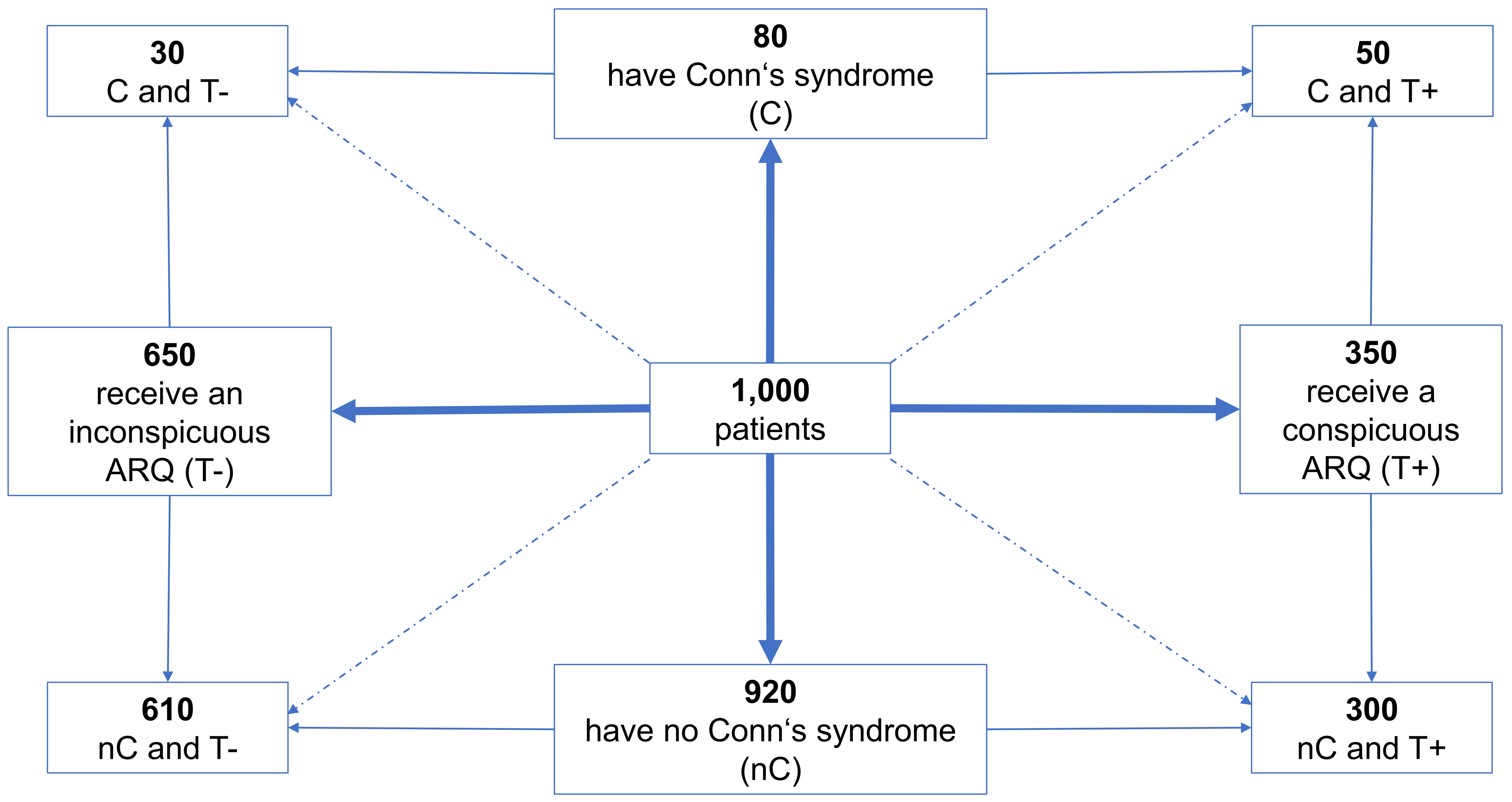

Supplement: S2 Fig — (TIF) [file pone.0283947.s006.tif]

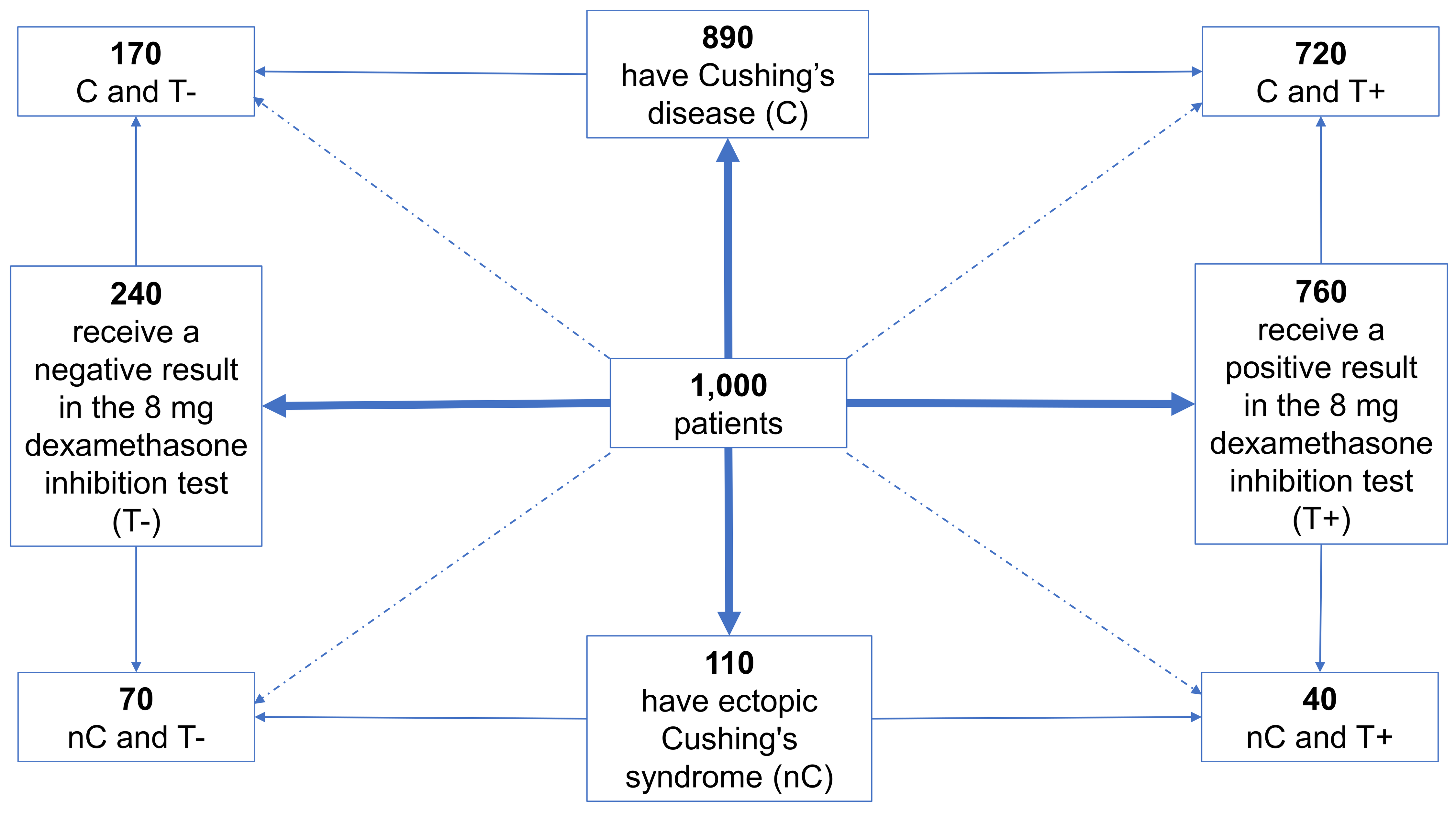

Supplement: S3 Fig — (TIF) [file pone.0283947.s007.tif]

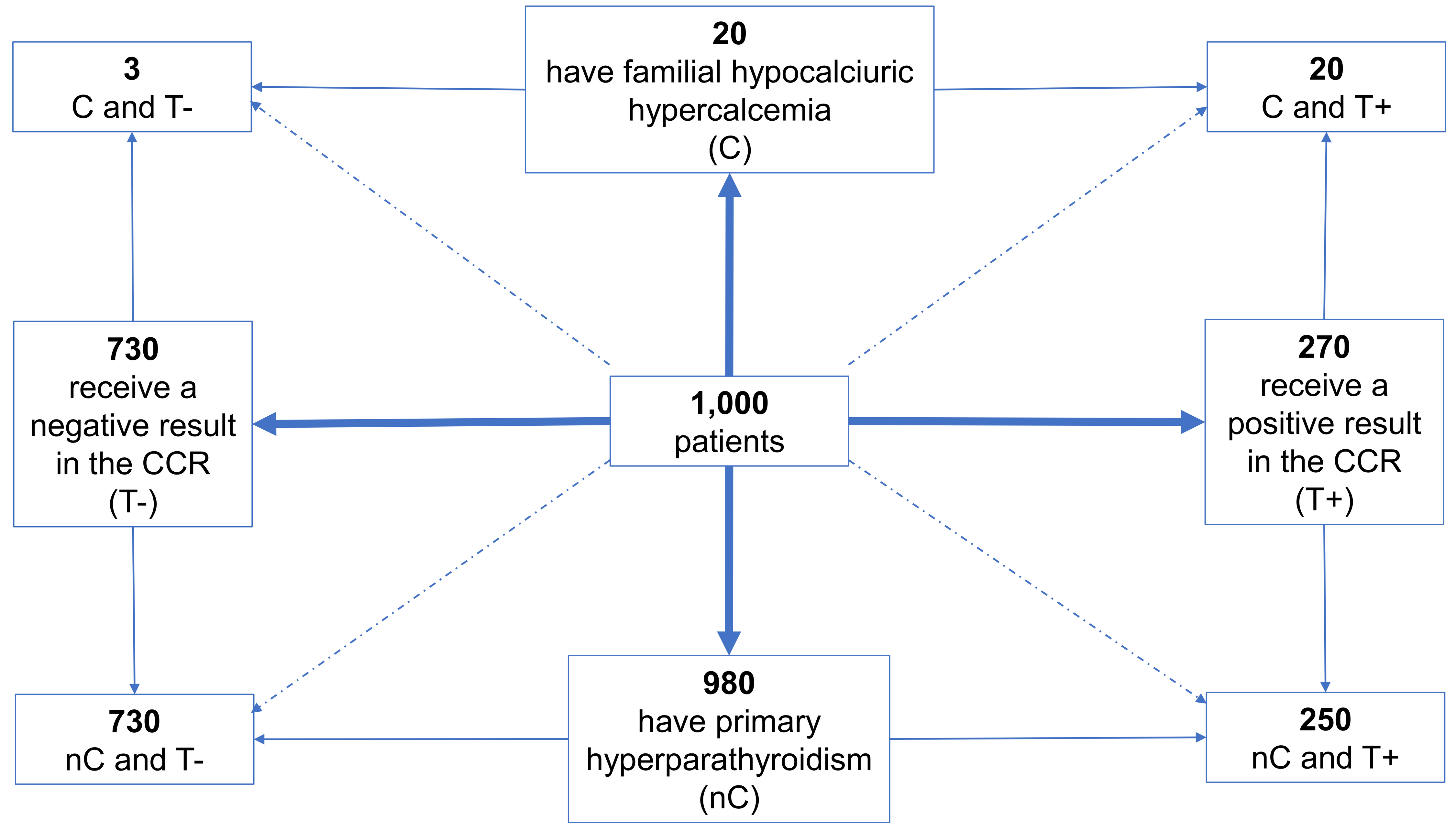

Supplement: S4 Fig — (TIF) [file pone.0283947.s008.tif]
